# Supplementary material for: Systematic Comparison of Temperature Effects on Antibody Performance via Automated Image Analysis: A Key for Primary Ciliary Dyskinesia Diagnostic
Source: Cells. 2025 Aug 11;14(16):1236. doi: 10.3390/cells14161236 (PMC12384470; doi:10.3390/cells14161236)

Supplementary Material

List of contents:

Supplementary Materials and Methods: 1

Supplementary Table S1: Antibodies used in the study 4

Supplementary Table S2: Correlations between the storage temperatures and analyzed image/ object parameters. 6

Supplementary Table S3: Overall performance of analyzed antibodies calculated on storage conditions excluding prolonged RT storage. 9

Supplementary Figure S1: Violin plots for the Distance_Centroid_mCilia_um values for various tested antibodies under different storage conditions. 10

Supplementary Figure S2: Influence of the fluorochrome color on the results of Cell Profiler analysis for various channels. 12

Supplementary Figure S3: Comparison of the values of the Distance_Centroid_mCilia for individual donors. 13

Supplementary Figure S4: IF staining of Donor 4 nasal cells before and after in vitro differentiation/ALI culture. 14

Supplementary Materials and Methods:

Immunofluorescence

Slides were stained as before [1]. Briefly, slides were fixed with 4% paraformaldehyde for 15 min and permeabilized with 0,2% Triton X-100/ PBS for 10 min at RT. Slides were blocked with blocking solution (10% BSA and 10% goat serum in 1x PBS) for 1 h at RT. Next, cells were incubated overnight in a moisture chamber at 4˚C with 0,15 ml of primary antibodies diluted in blocking solution (10% BSA and 10% goat serum in 1x PBS). Each slide was co-stained with one of the primary rabbit antibodies and the mouse AcTub antibody (Supplementary Table 1). The slides were observed using Leica DMi8 confocal microscope with LAS AF 2.7.3 software, with HC PL APO CS2 100x/ 1.4 OIL objective (Leica Microsystems GmbH, Wetzlar, Germany). Three lasers were used: 405 nm excitation with 420 to 550 nm detection range (DAPI), 499 nm excitation with 505 to 570 detection (AF488), 590 nm excitation with 600 to 750 nm detection range (AF594); laser intensity settings were adapted for each slide. Images of minimum 1024x1024 pix, were acquired for at least 30 cells using tile scan function in LAS AF software, raw images were exported to tiff files, with each channel saved as separate file.

Image analysis

All images were submitted to Cell Profiler software v. 4.2.7 [2] and analyzed using a custom pipeline (main pipeline steps in Figure 1). The Cell Profiler pipeline returns eight files (Experiment, Image, Nuclei,

Pairs_mTest, Test, mTest, Cilia, mCilia), see Zenodo repository under https://doi.org/10.5281/zenodo.15463498.

Experiment and Image files contain the description of the files analyzed, and the analysis parameters. Cilia, Test, and mCilia, mTest files contain the measurements of the objects detected in the green/red channels before or after the object merging steps in the pipeline **(Figure 1)**, respectively. Each measurement file contains the data for all identified objects, and the Children_mTest_Count parameter indicates whether the object has a pair in the other fluorescence channel (0- no pair, >0- paired). The Pairs_mTest file contains the information on the location of objects in the red channel (children) which overlap/pair with objects in the green channel (parents). To assure that the analyzed objects from various channels were present in the same cell, only the objects containing only 1 pair were taken for the parameter analysis.

Out of the 114 parameters measured by the CellProfiler pipeline, 81 parameters were found significantly different between paired objects from positive and negative control samples (p<0.001) (**Supplementary Table 2**). The majority of them characterized red channel objects in terms of their area & shape, and intensity (29 and 43 parameters, respectively). The second type of parameters described comparisons of the red-versus-green channel, preceded by the definition of paired objects from both channels (seven estimated correlation between the channels, and one measured distance between the centroids of paired objects) (Supplementary Table 1). Out of this group of significantly different parameters, we have chosen the representatives of each functional group (area/shape, intensity, channel correlation and distance). In the Area/Shape parameters group, we have chosen object area, as well as two parameters, which characterize the shape of the objects, i.e. area eccentricity and area compactness. Area Eccentricity characterizes how elongated or elliptical an object is, eccentricity values closer to 1 indicate objects that are more elliptical/stretched out, while values closer to 0 indicate more circular shapes. Area Compactness characterizes how "filled" and regular an object is relative to a circle with the same perimeter, compactness values higher than 1 indicate more irregular borders of the object.

Since the laser intensity settings during the image acquisition were not uniform for all slides, in the Intensity group, we only selected parameters not directly related to the object intensity (e.g. mean intensity, minimal intensity, etc), but characterize its distribution within the object, e.g. intensity mass displacement. Due to the same reason, in the group of 11 Correlation measurements, we have focused on the correlation calculated using the rank-weighted colocalization method (Correlation RWC), as it is a method less sensitive to the differences in overall object intensity levels. Moreover, we have decided to use also the Distance_Centroid_mCilia parameter, which describes the distance between geometric centers of overlapping (paired) objects in the red (tested antibodies) and the green channels (axoneme marker). This measurement is based purely on the spatial locations of the centroids and does not take

into account their pixel intensities. This parameter is only calculated when the localization of the object in the red channel (tested antibody) overlaps (pairs) with the object detected in the green channel (anti-Ac-αTub).

Statistical Analysis

Distribution of the values in CP profiler results was assessed using D'Agostino & Pearson, Anderson-Darling, Shapiro-Wilk and Kolmogorov-Smirnov distribution tests. Statistical significance of the differences between the storage condition groups and antibodies was assessed in GraphPad using non-parametric Kruskal-Wallis test with Dunn correction for multiple comparisons. Significance of the differences between mean distance to axoneme marker for CCDC39 and other antibodies was assessed in GraphPad Prism using t-test.

Supplementary Table S1: Antibodies used in the study

Antibody and serial number	Target	Provider, Cat#, RRID	Dilution	Reference
PRIMARY antibodies				
rabbit anti-DNAH5	ODA	Atlas Antibodies Cat#HPA037470, RRID:AB_10672348	1:800	[3]
rabbit anti-DNALI1	IDA	Atlas Antibodies Cat# HPA028305, RRID:AB_10601807	1:1000	[4–6]
rabbit anti-RSPH9	RS	Atlas Antibodies Cat#HPA031703, RRID:AB_10602126	1:400	[3,7,8]
rabbit anti- RSPH4a	RS	Atlas Antibodies Cat#HPA031196, RRID:AB_10601612	1:400	[3–5,7,9]
rabbit anti-GAS8	N-DRC	Atlas Antibodies Cat#HPA041311, RRID:AB_2677402	1:600	[3,10]
rabbit anti-CCDC39	MR	Atlas Antibodies Cat#HPA035364, RRID:AB_10601095	1:500	[11]
rabbit anti-CCDC40	MR	Atlas Antibodies Cat#HPA022974, RRID:AB_1848791	1:1600	[6]
rabbit anti- Spef2	CP	Atlas Antibodies Cat#HPA039606, RRID:AB_10671044	1:300	[9,12,13]
mouse anti-acetylated α-tubulin (Ac-αTub)	MTs, axonemal marker	Sigma-Aldrich Cat# T6793, RRID:AB_477585	1:10,000	[5,14]
SECONDARY antibodies				
AlexaFluor 594 goat anti-rabbit IgG (H+L)	Tested antibodies (red)	A11037, Invitrogen	1:2,500	[1,15]
AlexaFluor 488 goat anti-mouse IgG (H+L)	Ac-αTub (green)	A11029, Invitrogen	1:2,500	[1,15]

Supplementary References:

1. Bukowy-Bieryłło, Z.; Daca-Roszak, P.; Jurczak, J.; Przystałowska-Macioła, H.; Jaksik, R.; Witt, M.; Ziętkiewicz, E. In Vitro Differentiation of Ciliated Cells in ALI-Cultured Human Airway Epithelium - The Framework for Functional Studies on Airway Differentiation in Ciliopathies. *Eur J Cell Biol* **2022**, *101*, 151189, doi:10.1016/j.ejcb.2021.151189.
2. Stirling, D.R.; Swain-Bowden, M.J.; Lucas, A.M.; Carpenter, A.E.; Cimini, B.A.; Goodman, A. CellProfiler 4: Improvements in Speed, Utility and Usability. *BMC Bioinformatics* **2021**, *22*, 433, doi:10.1186/s12859-021-04344-9.
3. Shoemark, A.; Frost, E.; Dixon, M.; Ollosson, S.; Kilpin, K.; Patel, M.; Scully, J.; Rogers, A.V.; Mitchison, H.M.; Bush, A.; et al. Accuracy of Immunofluorescence in the Diagnosis of Primary Ciliary Dyskinesia. *Am J Respir Crit Care Med* **2017**, *196*, 94–101, doi:10.1164/rccm.201607-1351OC.
4. Müller, L.; Savas, S.T.; Tschanz, S.A.; Stokes, A.; Escher, A.; Nussbaumer, M.; Bullo, M.; Kuehni, C.E.; Blanchon, S.; Jung, A.; et al. A Comprehensive Approach for the Diagnosis of Primary Ciliary Dyskinesia—Experiences from the First 100 Patients of the PCD-UNIBE Diagnostic Center. *Diagnostics* **2021**, *11*, doi:10.3390/diagnostics11091540.

5. Onoufriadis, A.; Shoemark, A.; Schmidts, M.; Patel, M.; Jimenez, G.; Liu, H.; Thomas, B.; Dixon, M.; Hirst, R.A.; Rutman, A.; et al. Targeted NGS Gene Panel Identifies Mutations in RSPH1 Causing Primary Ciliary Dyskinesia and a Common Mechanism for Ciliary Central Pair Agenesis Due to Radial Spoke Defects. *Human Molecular Genetics* **2014**, *23*, 3362–3374, doi:10.1093/hmg/ddu046.
6. Liu, Z.; Nguyen, Q.P.H.; Guan, Q.; Albulescu, A.; Erdman, L.; Mahdaviyeh, Y.; Kang, J.; Ouyang, H.; Hegele, R.G.; Moraes, T.; et al. A Quantitative Super-Resolution Imaging Toolbox for Diagnosis of Motile Ciliopathies. *Science Translational Medicine* **2020**, *12*, eaay0071, doi:10.1126/scitranslmed.aay0071.
7. Frommer, A.; Hjeij, R.; Loges, N.T.; Edelbusch, C.; Jahnke, C.; Raidt, J.; Werner, C.; Wallmeier, J.; Große-Onnebrink, J.; Olbrich, H.; et al. Immunofluorescence Analysis and Diagnosis of Primary Ciliary Dyskinesia with Radial Spoke Defects. *Am J Respir Cell Mol Biol* **2015**, *53*, 563–573, doi:10.1165/rcmb.2014-0483OC.
8. Al-Mutairi, D.A.; Alsabah, B.H.; Pennekamp, P.; Omran, H. Mapping the Most Common Founder Variant in RSPH9 That Causes Primary Ciliary Dyskinesia in Multiple Consanguineous Families of Bedouin Arabs. *Journal of Clinical Medicine* **2023**, *12*, doi:10.3390/jcm12206505.
9. Burgoyne, T.; Fassad, M.R.; Schultz, R.; Elenius, V.; Lim, J.S.Y.; Freke, G.; Rai, R.; Mohammed, M.A.; Mitchison, H.M.; Sironen, A.I. HYDIN Variants Cause Primary Ciliary Dyskinesia in the Finnish Population. *Pediatric Pulmonology* **2024**, *59*, 3601–3609, doi:10.1002/ppul.27267.
10. Olbrich, H.; Cremers, C.; Loges, N.T.; Werner, C.; Nielsen, K.G.; Marthin, J.K.; Philipsen, M.; Wallmeier, J.; Pennekamp, P.; Menchen, T.; et al. Loss-of-Function GAS8 Mutations Cause Primary Ciliary Dyskinesia and Disrupt the Nexin-Dynein Regulatory Complex. *Am J Hum Genet* **2015**, *97*, 546–554, doi:10.1016/j.ajhg.2015.08.012.
11. Merveille, A.-C.; Davis, E.E.; Becker-Heck, A.; Legendre, M.; Amirav, I.; Bataille, G.; Belmont, J.; Beydon, N.; Billen, F.; Clément, A.; et al. CCDC39 Is Required for Assembly of Inner Dynein Arms and the Dynein Regulatory Complex and for Normal Ciliary Motility in Humans and Dogs. *Nat Genet* **2011**, *43*, 72–78, doi:10.1038/ng.726.
12. Cindrić, S.; Dougherty, G.W.; Olbrich, H.; Hjeij, R.; Loges, N.T.; Amirav, I.; Philipsen, M.C.; Marthin, J.K.; Nielsen, K.G.; Sutharsan, S.; et al. SPEF2- and HYDIN-Mutant Cilia Lack the Central Pair-Associated Protein SPEF2, Aiding Primary Ciliary Dyskinesia Diagnostics. *Am J Respir Cell Mol Biol* **2020**, *62*, 382–396, doi:10.1165/rcmb.2019-0086OC.
13. Mori, M.; Kido, T.; Sakamoto, N.; Ozasa, M.; Kido, K.; Noguchi, Y.; Tokito, T.; Okuno, D.; Yura, H.; Hara, A.; et al. Novel SPEF2 Variant in a Japanese Patient with Primary Ciliary Dyskinesia: A Case Report and Literature Review. *Journal of Clinical Medicine* **2023**, *12*, 317, doi:10.3390/jcm12010317.
14. Omran, H.; Kobayashi, D.; Olbrich, H.; Tsukahara, T.; Loges, N.T.; Hagiwara, H.; Zhang, Q.; Leblond, G.; O'Toole, E.; Hara, C.; et al. Ktu/PF13 Is Required for Cytoplasmic Pre-Assembly of Axonemal Dyneins. *Nature* **2008**, *456*, 611–616, doi:10.1038/nature07471.
15. Omran, H.; Loges, N.T. Immunofluorescence Staining of Ciliated Respiratory Epithelial Cells. *Methods Cell Biol* **2009**, *91*, 123–133, doi:10.1016/S0091-679X(08)91007-4.

Supplementary Table S2: Correlations between the storage temperatures and analyzed image/ object parameters.

Names of the columns contain the measurement group (AreaShape, Intensity, Correlation etc), followed by the name of the parameter measured (e.g. Area, Eccentricity etc). Names of the columns containing only the measurement group and the parameter name refer to measurements performed on the green channel. Column names with suffix "Mean_mTest" refer to the measurements done on the red channel. Parameters chosen for further analyses are shown in bold (column 1). P-values lower than 0.001 are shown in bold (column 3).

Parameter	Rho	P_value
**Mean_mTest_Correlation_RWC_RED_GREEN**	-0.115661134163905	**1.4731882347589e-67**
Mean_mTest_Correlation_Manders_RED_GREEN	-0.111155174785092	**1.66073648270303e-62**
Mean_mTest_Intensity_MassDisplacement_GREEN	0.110559925050577	**7.45421277797541e-62**
Mean_mTest_Intensity_LowerQuartileIntensity_GREEN	-0.095947036415811	**5.85253404288507e-47**
Intensity_MeanIntensityEdge_RED	0.0873069756130233	**3.74086142571518e-39**
Intensity_MinIntensity_RED	0.0852235041321796	**2.21063353004736e-37**
Intensity_MinIntensityEdge_RED	0.0851610998501053	**2.49411337507631e-37**
Intensity_MaxIntensityEdge_RED	0.083886178816177	**2.87750123785879e-36**
Mean_mTest_Intensity_MedianIntensity_GREEN	-0.0838493629037626	**3.08636498961121e-36**
Intensity_StdIntensityEdge_RED	0.0817099968279706	**1.71734278255422e-34**
Mean_mTest_Intensity_MaxIntensity_RED	0.0814502192120748	**2.77794435987991e-34**
Mean_mTest_Intensity_IntegratedIntensityEdge_RED	0.0797727878882791	**5.97584730214557e-33**
**Mean_mTest_AreaShape_Eccentricity**	-0.0745026468670747	**6.07065770029549e-29**
**Mean_mTest_AreaShape_Compactness**	0.0739983777426539	**1.42011102755787e-28**
Mean_mTest_AreaShape_FormFactor	-0.073998375987077	**1.42011521504808e-28**
Intensity_LowerQuartileIntensity_RED	0.0734700337700935	**3.43837580785071e-28**
**Mean_mTest_Intensity_MassDisplacement_RED**	0.0709328993172888	**2.19970517734353e-26**
Intensity_MedianIntensity_RED	0.0705986203437883	**3.76368199691447e-26**
Mean_mTest_AreaShape_MinorAxisLength	0.068394778517376	**1.21901537395203e-24**
Intensity_MeanIntensity_RED	0.0682695941226386	**1.48039695911839e-24**
Mean_mTest_AreaShape_MinFeretDiameter	0.0681015125610288	**1.92051257338006e-24**
AreaShape_Area	-0.0675331148691324	**4.60927906596462e-24**
Mean_mTest_Correlation_Overlap_GREEN_RED	-0.0667691266729029	**1.47812779039696e-23**
Intensity_MinIntensityEdge_GREEN	0.0663331307321573	**2.85738363414516e-23**
Mean_mTest_AreaShape_MaximumRadius	0.0648248365524599	**2.70358674180827e-22**
Mean_mTest_Intensity_MeanIntensity_GREEN	-0.0647380814496224	**3.07186379928636e-22**
Intensity_UpperQuartileIntensity_RED	0.0643785951368104	**5.2051799925484e-22**
Mean_mTest_AreaShape_Perimeter	0.0641630917112213	**7.13069689497273e-22**
Mean_mTest_Intensity_IntegratedIntensity_RED	0.0633740560622559	**2.23750212610428e-21**
**Mean_mTest_Distance_Centroid_mCilia**	0.0626689364997743	**6.14390647972798e-21**
AreaShape_MajorAxisLength	-0.0597616850928501	**3.51598881603417e-19**
Intensity_MinIntensity_GREEN	0.0579942240969737	**3.7533998249285e-18**
Mean_mTest_AreaShape_EulerNumber	-0.0576423867542222	**5.96372981612822e-18**
AreaShape_MinorAxisLength	-0.0562887158860364	**3.45154428691022e-17**
AreaShape_MaxFeretDiameter	-0.0548696608807477	**2.08099471003424e-16**
Mean_mTest_AreaShape_BoundingBoxMinimum_X	-0.0548510547286008	**2.12996224180127e-16**
Intensity_MaxIntensity_RED	0.0546908662299549	**2.60133675927785e-16**

Mean_mTest_Intensity_UpperQuartileIntensity_GREEN	-0.052940785557876	**2.22635104016063e-15**
Mean_mTest_AreaShape_ConvexArea	0.0528858975100879	**2.37879766369995e-15**
Mean_mTest_Intensity_MinIntensityEdge_RED	0.0527225505351725	**2.89591991616328e-15**
AreaShape_MinFeretDiameter	-0.052585927370506	**3.41225304014872e-15**
Mean_mTest_Intensity_MinIntensityEdge_GREEN	-0.0521190663563624	**5.95891986688447e-15**
**Mean_mTest_AreaShape_Area**	0.05205459139912	**6.43339864513345e-15**
Mean_mTest_AreaShape_EquivalentDiameter	0.0520545399707035	**6.43379157048685e-15**
Mean_mTest_AreaShape_BoundingBoxArea	0.0509127093002062	**2.46075771011882e-14**
Mean_mTest_Intensity_MeanIntensityEdge_GREEN	-0.0502886572747398	**5.06015969494861e-14**
AreaShape_Perimeter	-0.0499470451551088	**7.48108827749426e-14**
Mean_mTest_Intensity_MinIntensity_GREEN	-0.0495536753389163	**1.16976361187347e-13**
AreaShape_EulerNumber	0.0485424087238789	**3.63352377033922e-13**
Mean_mTest_Intensity_StdIntensityEdge_RED	-0.0470784320080157	**1.80081495553667e-12**
Mean_mTest_AreaShape_BoundingBoxMinimum_Y	-0.046183348807341	**4.68089098183343e-12**
Intensity_UpperQuartileIntensity_GREEN	0.0442933552846822	**3.31925637184409e-11**
Mean_mTest_Location_MaxIntensity_X_GREEN	-0.0435784351882528	**6.82189349171669e-11**
Mean_mTest_AreaShape_Center_X	-0.0433915942188394	**8.21983837019178e-11**
Intensity_MADIntensity_RED	0.0433576427917249	**8.50234174526413e-11**
Mean_mTest_Location_CenterMassIntensity_X_RED	-0.0432470193119049	**9.49026271564208e-11**
Mean_mTest_Location_MaxIntensity_X_RED	-0.0430592735917971	**1.14296222797793e-10**
Mean_mTest_Intensity_MinIntensity_RED	0.0430049098018489	**1.20601289281675e-10**
Mean_mTest_Location_CenterMassIntensity_X_GREEN	-0.0426035200298296	**1.78919578623249e-10**
Intensity_MeanIntensity_GREEN	0.0417880900271573	**3.94386409917333e-10**
Mean_mTest_AreaShape_Solidity	-0.0417533884733877	**4.07745217281211e-10**
Mean_mTest_Correlation_RWC_GREEN_RED	-0.0414159693658734	**5.62933921704592e-10**
Mean_mTest_Intensity_MADIntensity_GREEN	-0.0412655604906338	**6.4944767114122e-10**
Mean_mTest_AreaShape_MaxFeretDiameter	0.0406750110791186	**1.13298733305405e-09**
Mean_mTest_Correlation_Costes_RED_GREEN	-0.0385137101883461	**8.13506572571191e-09**
Intensity_MedianIntensity_GREEN	0.0381943449244061	**1.07918100973505e-08**
Correlation_Overlap_GREEN_RED	-0.0373095094140293	**2.33384485994372e-08**
Mean_mTest_Intensity_StdIntensity_GREEN	0.0371274733314242	**2.72937650226708e-08**
Mean_mTest_Correlation_Costes_GREEN_RED	-0.0367984971700827	**3.6152585605092e-08**
Mean_mTest_AreaShape_MeanRadius	0.0354448544818111	**1.12108654845955e-07**
Correlation_RWC_RED_GREEN	-0.0353938965663508	**1.1689672588802e-07**
Intensity_StdIntensity_GREEN	0.0351319216284991	**1.44807549248052e-07**
Mean_mTest_Location_CenterMassIntensity_Y_GREEN	-0.0349418979122752	**1.68979509999105e-07**
Intensity_MassDisplacement_RED	-0.03485817340441	**1.80827572656922e-07**
Mean_mTest_Location_MaxIntensity_Y_GREEN	-0.0343282580007987	**2.76694482502182e-07**
Mean_mTest_Location_CenterMassIntensity_Y_RED	-0.0338470463007074	**4.05001749894641e-07**
Mean_mTest_AreaShape_Center_Y	-0.0338181061082394	**4.14321741439672e-07**
Intensity_MassDisplacement_GREEN	-0.0330454271369436	**7.554798045844e-07**
Intensity_IntegratedIntensity_GREEN	-0.0326780597590011	**1.00065336177349e-06**
Mean_mTest_Location_MaxIntensity_Y_RED	-0.0325549017497455	**1.09880113624653e-06**
Intensity_LowerQuartileIntensity_GREEN	0.0322175373480849	**1.41742124739326e-06**
Mean_mTest_AreaShape_BoundingBoxMaximum_X	-0.0318255701059463	**1.89945944178851e-06**
Intensity_IntegratedIntensityEdge_RED	0.0290745932645528	**1.34979854771562e-05**
Mean_mTest_Intensity_StdIntensity_RED	0.0290049336438959	**1.41551084515568e-05**

Intensity_MeanIntensityEdge_GREEN	0.0288940343984262	**1.52646045089015e-05**
Intensity_StdIntensity_RED	0.0288407960591754	**1.58262281187249e-05**
Intensity_MADIntensity_GREEN	0.0276190266137396	**3.56630114366653e-05**
AreaShape_Extent	-0.0271019179725907	**4.98155437207045e-05**
Mean_mTest_AreaShape_MajorAxisLength	0.0268328229207534	**5.91442660797361e-05**
Mean_mTest_Correlation_Correlation_GREEN_RED	-0.0242258164603437	**0.000287984490430112**
Correlation_Correlation_GREEN_RED	-0.0239038606181855	**0.000346665205593237**
Mean_mTest_Intensity_MaxIntensity_GREEN	0.0233843661033745	**0.000465432217230539**
Intensity_MaxIntensity_GREEN	0.0229615480818657	**0.000589062772197361**
Mean_mTest_AreaShape_BoundingBoxMaximum_Y	-0.0227881577322848	**0.000648098259255224**
Intensity_IntegratedIntensityEdge_GREEN	-0.022195163565802	**0.000894165885623938**
Intensity_StdIntensityEdge_GREEN	-0.0221531136936755	**0.000914550465971032**
Mean_mTest_Intensity_MeanIntensityEdge_RED	0.0209522315742683	0.00171384826553063
AreaShape_Solidity	-0.0192271807084375	0.00400746437963143
Mean_mTest_AreaShape_Extent	-0.0184735492135954	0.00569639697011823
Mean_mTest_AreaShape_MedianRadius	0.0169250697204568	0.0113096471185149
Mean_mTest_Intensity_MedianIntensity_RED	-0.0158943917498985	0.0173732006003841
Mean_mTest_Correlation_K_RED_GREEN	-0.0158744478057185	0.0175143787030025
Mean_mTest_Intensity_MaxIntensityEdge_GREEN	0.0152033368700986	0.0228891846375894
AreaShape_Compactness	-0.0151555623294804	0.0233213676682738
AreaShape_FormFactor	0.0151555623294804	0.0233213676682738
Mean_mTest_Intensity_IntegratedIntensity_GREEN	0.0140226254031119	0.0358557148246747
Intensity_IntegratedIntensity_RED	-0.0129660589300417	0.0523280229893498
Mean_mTest_Intensity_MaxIntensityEdge_RED	0.0118833783332502	0.0753405397976296
Mean_mTest_Correlation_K_GREEN_RED	0.0106770412020535	0.110078022545708
Mean_mTest_Intensity_StdIntensityEdge_GREEN	0.0099257964788003	0.137434881265135
Mean_mTest_Correlation_Manders_GREEN_RED	0.0085077506460563	0.202950547026143
Mean_mTest_Intensity_LowerQuartileIntensity_RED	-0.00790921666355896	0.236564971341632
Mean_mTest_Intensity_IntegratedIntensityEdge_GREEN	0.00757717244794935	0.256826272244064
AreaShape_Eccentricity	-0.0073334098797523	0.272447220805256
Mean_mTest_Intensity_MADIntensity_RED	-0.00477117033372176	0.475227315149796
Mean_mTest_AreaShape_Orientation	-0.00388568121761845	0.560912743905971
Mean_mTest_Intensity_UpperQuartileIntensity_RED	-0.00354038895209794	0.596241241856369
Intensity_MaxIntensityEdge_GREEN	-0.00290160791932087	0.664128324904755
Correlation_RWC_GREEN_RED	0.000516425948932618	0.938399150872506
Mean_mTest_Intensity_MeanIntensity_RED	-0.000252977733007192	0.969801225317864

Supplementary Table S3: Overall performance of analyzed antibodies calculated on storage conditions excluding prolonged RT storage.

For each of the analyzed parameters, antibodies were ranked according to the mean parameter's value for all storage conditions (see rightmost columns in the heatmaps). The overall performance rank was calculated as the mean from all parameters. Colors of the bars below the table indicate the performance (green – very good, yellow – average, red – poor). Calculations were made for green objects pairing with only 1 red object.

	RSPH4A	DNAH5	GAS8	SPEF2	RSPH9	CCDC40	DNALI1	CCDC39
Mean_mTest_Correlation_RWC_RED_GREEN	1	2	7	4	6	5	3	8
Mean_mTest_AreaShape_Compactness	1	3	2	5	4	6	7	8
Mean_ mTest_AreaShape_Area	1	2	3	5	4	6	8	7
Mean_mTest_Area_Eccentricity	1	3	6	2	7	5	4	8
Mean_mTest_Intensity_Mass_Displacement	1	2	5	4	3	6	7	8
Mean_mTest_Distance_Centroid_mCilia	4	1	2	5	3	6	7	8
	1.5	2.2	4.2	4.2	4.5	5.7	6.0	7.8

Supplementary Figure S1: Violin plots for the Distance_Centroid_mCilia_um values for various tested antibodies under different storage conditions.

Violin plots for the optimal storage condition (−80oC/28d; “positive control”) for the analyzed antibodies revealed a weak dispersion of the distance values, the majority of them concentrated around the median, which was itself localized close to the 0 value. As expected, the “negative control“ (RT/28d) was significantly different, with much higher median and range of the distance values. A comparably deleterious effect was observed for slides stored at RT for 14 days, followed by 14 days at −80oC.

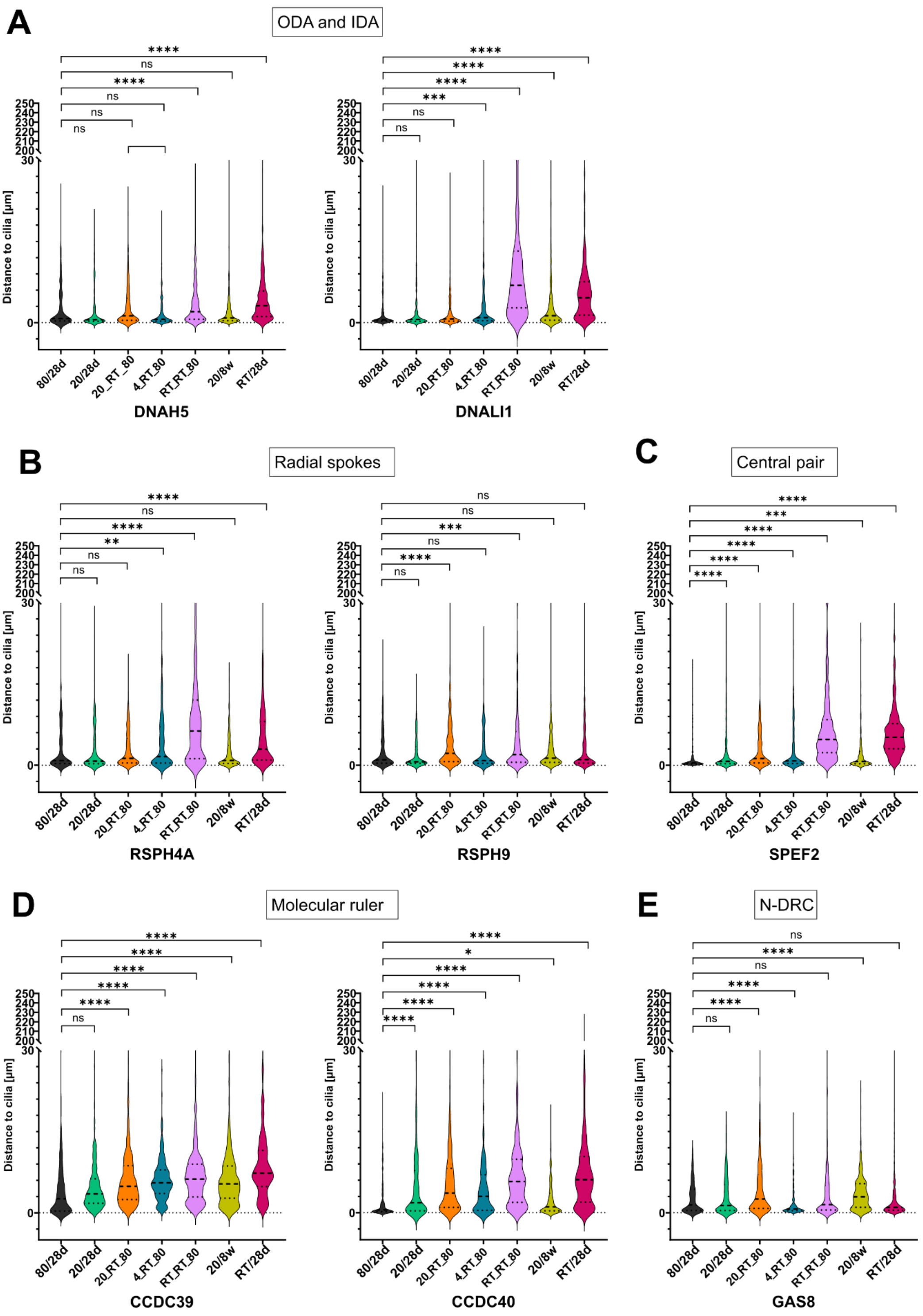

*Distribution of the distances between the centroids of paired red and green objects (Distance_Centroid_mCilia). A: ODA and IDA proteins; B: RS proteins; C:CP protein; D: MR proteins; E: N-DRC proteins. Thick dotted lines indicate median values, thin dotted lines indicate first and third quartile of the distance. Pairwise-comparisons between the centroid distance at the optimal storage conditions (-80oC for 28d) and other tested conditions were done using Kruskal-Wallis test with Dunn correction for multiple testing. *** indicates $p < 0.001$, * indicates* $p < 0.01$, $p < 0.05$, ns = not significant*).*

Supplementary Figure S2: Influence of the fluorochrome color on the results of Cell Profiler analysis for various channels.

Slides stored at −80oC_28d and RT_28d were co-stained for DNALI1 and axoneme marker, using secondary antibodies associated with fluorochromes of various colors (green line– Alexa Fluor 488, red line– Alexa Fluor 594). While the fluorochrome switch resulted in the switch of the corresponding values, the relative differences between the best and worst storage conditions remained similar.

Left panel: results for DNALI1 antibody; right panel: results for axoneme marker. The color of the line indicates the color of the fluorochrome used in the staining/ channel color. Numbers on the X axis indicate the storage conditions: best conditions (−80C_28d), worst conditions (RT_28d) or the mean value between the conditions (mean is shown only for informative purposes).

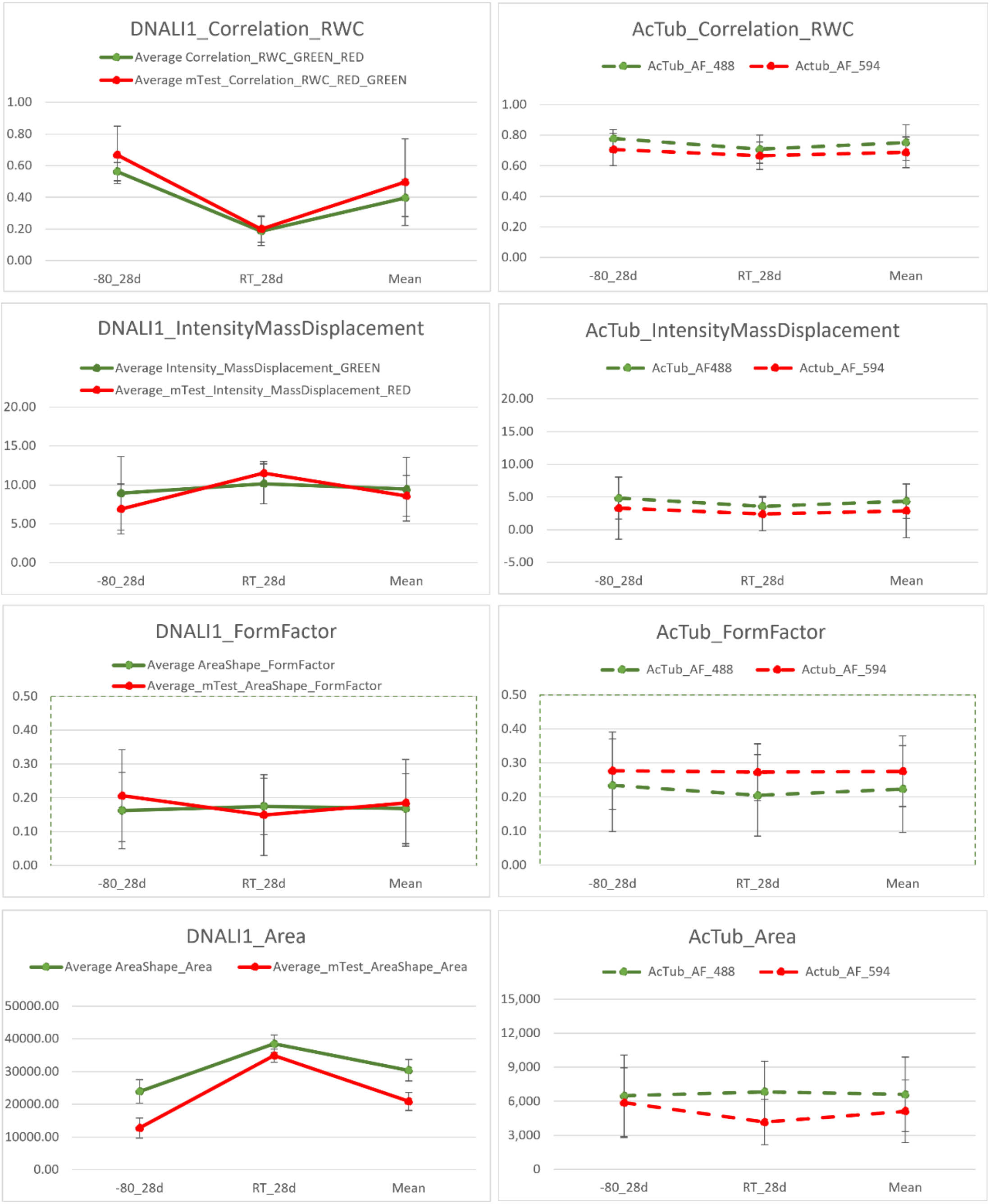

Supplementary Figure S3: Comparison of the values of the Distance_Centroid_mCilia for individual donors.

Data are expressed in um, points display the median values for individual donors +/- median absolute deviation (MAD). Grand median (purple) represents the median value from all 5 donors +/− MAD. Donor 4 (discrepant from other donors) is shown in yellow.

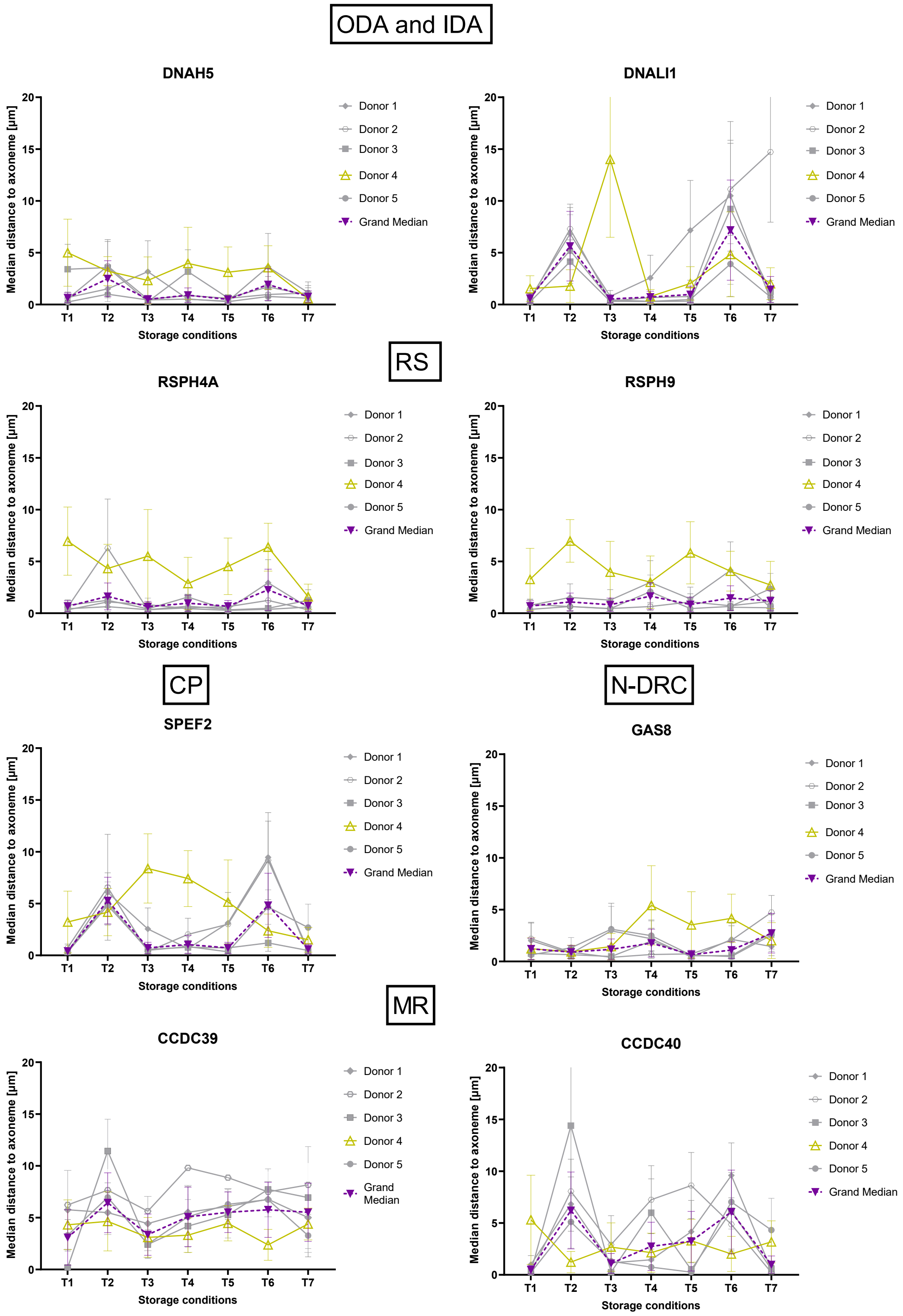

Supplementary Figure S4: IF staining of Donor 4 nasal cells before and after in vitro differentiation/ALI culture.

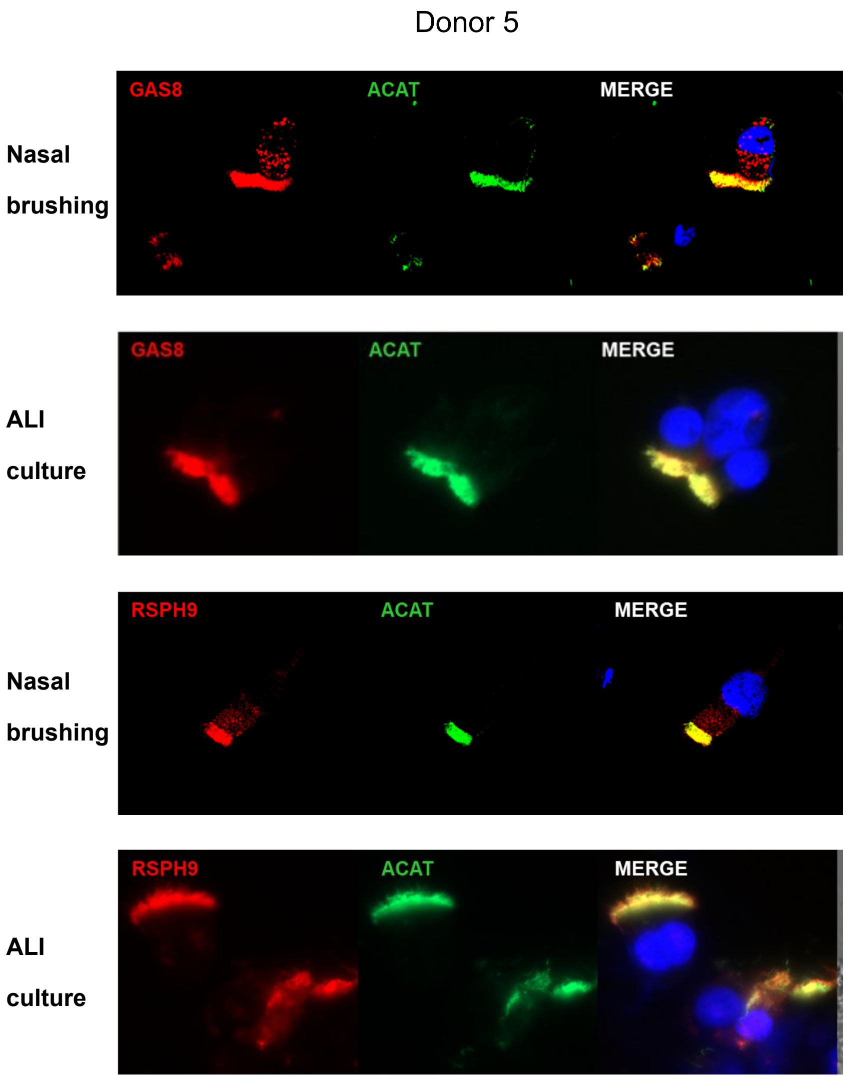

Supplement: Supplementary file 1 [file cells-14-01236-s001.zip › cells-3785937-supplementary_V3_clean.pdf]
